# Modified Linggui Zhugan Decoction protects against ventricular remodeling through ameliorating mitochondrial damage in post-myocardial infarction rats

**DOI:** 10.3389/fcvm.2022.1038523

**Published:** 2023-01-10

**Authors:** Mi Xiang, Xin Zhao, Yingdong Lu, Yang Zhang, Fan Ding, Lifei Lv, Yuling Wang, Zihuan Shen, Li Li, Xiangning Cui

**Affiliations:** ^1^Department of Cardiovascular, Guang’anmen Hospital, China Academy of Chinese Medical Sciences, Beijing, China; ^2^Department of Pathology, Guang’anmen Hospital, China Academy of Chinese Medical Sciences, Beijing, China; ^3^First Clinical Medical School, Shandong University of Chinese Medicine, Shandong, China

**Keywords:** Modified Linggui Zhugan Decoction, myocardial infarction, ventricular remodeling, mitochondrial damage, apoptosis

## Abstract

**Introduction:**

Modified Linggui Zhugan Decoction (MLZD) is a Traditional Chinese Medicine prescription developed from Linggui Zhugan Decoction (LZD) that has been used for the clinical treatment of ischemic cardiovascular diseases. However, the cardioprotective mechanism of MLZD against post-myocardial infarction (MI) ventricular remodeling remains unclear.

**Methods:**

We explored the effects of MLZD on ventricular remodeling and their underlying mechanisms, respectively, in SD rats with MI models and in H9c2 cardiomyocytes with oxygen-glucose deprivation (OGD) models. The cardiac structure and function of rats were measured by echocardiography, HE staining, and Masson staining. Apoptosis, inflammation, mitochondrial structure and function, and sirtuin 3 (SIRT3) expression were additionally examined.

**Results:**

MLZD treatment significantly ameliorated cardiac structure and function, and thus reversed ventricular remodeling, compared with the control. Further research showed that MLZD ameliorated mitochondrial structural disruption, protected against mitochondrial dynamics disorder, restored impaired mitochondrial function, inhibited inflammation, and thus inhibited apoptosis. Moreover, the decreased expression level of SIRT3 was enhanced after MLZD treatment. The protective effects of MLZD on SIRT3 and mitochondria, nevertheless, were blocked by 3-TYP, a selective inhibitor of SIRT3.

**Discussion:**

These findings together revealed that MLZD could improve the ventricular remodeling of MI rats by ameliorating mitochondrial damage and its associated apoptosis, which might exert protective effects by targeting SIRT3.

## 1. Introduction

Coronary artery disease (CAD) remains the leading cause of death in developed as well as developing countries (1), among which myocardial infarction (MI), one of the worst heart diseases (2), accounts for the majority of CAD deaths (1). Although 85% of the estimated 800,000 Americans who suffer MI each year reportedly survive, these survivors are left with cardiac dysfunction and a shortened life expectancy (3). Cardiac remodeling is caused or exacerbated by a series of pathological changes after MI, manifests pathologically as myocardial hypertrophy and fibrosis, and results in cardiac dysfunction, heart failure, malignant arrhythmia, and even cardiac death (1, 4, 5). Despite the widespread clinical use of multiple techniques, there is still a need for more effective treatments (2).

Mitochondria are more commonly found in cardiomyocytes than in other mammalian cells (6), and have been investigated as therapeutic targets in myocardial infarction (7). Ischemic oxidative damage leads to mitochondrial Ca^2+^ bursts, non-selective mitochondrial permeability transition pore (MPTP) opening, and mitochondrial membrane potential (MMP) collapse (8). These mitochondrial changes above will lead to the release of pro-apoptotic factors and thus apoptosis (9, 10). Apoptosis is closely associated with LV remodeling and heart failure following acute myocardial infarction and is a potential target for therapeutic intervention (11). Mitochondrial fusion/fission, also described as the mitochondrial dynamics that change rapidly in response to external damage and metabolic status, is crucial to maintaining mitochondrial homeostasis (12). Mitochondrial dynamics exert a significant influence on the process of myocardial infarction, cardiac hypertrophy, and heart failure (13–15).

Traditional Chinese Medicine (TCM) is regarded as complementary and alternative medicine for the primary and secondary prevention of cardiovascular diseases (16). Linggui Zhugan Decoction (LZD) is a well-known TCM formula that contains Poria, Ramulus cinnamomi, Rhizoma atractylodis macrocephalae, and Radix Glycyrrhizae, which was documented in Jin Gui Yao Lue, a classical work of Zhongjing Zhang in the Han dynasty. LZD is deemed as one of the effective and mild classic prescriptions for applying in the clinical treatment of heart failure, and its efficacy has been shown through clinical studies in HF patients (17–19). Modern pharmacological studies revealed that LZD could improve the structure and function of the heart, and reverse the pathological progression of cardiac hypertrophy to heart failure (17, 20). The Modified Linggui Zhugan Decoction (MLZD) was modified from LZD, consisting of Radix astragali [*Astragalus membranaceus* (Fisch.) Bge.], Panax ginseng [*Panax ginseng* C. A. Mey.], Ramulus cinnamomi [*Cinnamomum cassia* Presl], Poria [*Poria cocos* (Schw.) Wolf], Rhizoma atractylodis macrocephalae [*Atractylodes macrocephala* Koidz.], Rhizoma alismatis [*Alisma orientalis* (Sam.) Juzep.], Radix salviae miltiorrhizae [*Salvia miltiorrhiza* Bunge.], Pericarpium areca [*Areca catechu* L.], Semen lepidii [*Lepidium apetalum* Willd.], and Radix angelicae sinensis [*Angelica sinensis* (Oliv.) Diels], which exerts crucial protections on the heart. For example, an extract of Radix astragali, calycosin, was reported to inhibit neutrophil infiltration and protect heart integrity in isoproterenol-induced MI by synergizing with gallic acid (21). The cardioprotective effects of Panax ginseng or ginsenosides have been reported, through preventing MI and heart failure (22). The ethyl acetate extract of Ramulus cinnamomi and its bioactive substance cinnamic acid play a protective role in myocardial ischemia/reperfusion injury (23, 24). Cinnamaldehyde, another core active ingredient of Ramulus cinnamomi (25, 26), was revealed to protect against MI injury as a transient receptor potential ankyrin 1 agonist (27), and to protect rats from cardiac inflammation and fibrosis through inhibiting Nod-like receptor pyrin domain 3 (NLRP3) inflammasome activation (28). Atractylenolide I, an active ingredient isolated from Rhizoma atractylodis macrocephalae, protects against myocardial ischemia/reperfusion injury by attenuating mitochondrial dysfunction and caspase-3 activity (29).

Nevertheless, what remains to be further explored is whether MLZD can protect against ventricular remodeling after MI, and the mechanisms through which it acts. Investigating these important questions may be helpful in providing a promising therapy for these heart diseases. In this work, we investigated the underlying mechanisms of MLZD in ventricular remodeling, respectively using SD rats with MI models and H9c2 cells with OGD models, seeking to verify our hypothesis that MLZD exerts pharmacological effects targeting mitochondrial damage and apoptosis, thereby alleviating post-MI ventricular remodeling.

## 2. Materials and methods

### 2.1. Screening of MLZD ingredients

Modified Linggui Zhugan Decoction ingredients were screened via network pharmacology and qualitatively analyzed via liquid chromatography-mass spectrometry (LC-MS) analysis. From the Traditional Chinese Medicine System Pharmacology (TCMSP) database,^1^ the chemical constituents of 10 traditional Chinese medicines in MLZD were sequentially retrieved. Referring to the screening conditions of TCMSP, that is, oral bioavailability (OB) ≥ 30% and drug-likeness (DL) ≥ 0.18, eligible potential active ingredients were obtained. LC-MS analysis was subsequently performed. A hundred milligrams of MLZD powder was dissolved in water (5 ml) with the assistance of ultrasound. Then the solution was filtered. The 50 μl of the filtrate was diluted with MeCN to 1 ml, then it was analyzed by LC-Mass (Waters Acquity ultra-performance LC). The data was collected from the spectrum of positive charges.

### 2.2. Model induction of MI in rats

Male SD rats (200–220 g) were obtained from Beijing Huafukang Biotechnology Co., Ltd (Animal license number: SCXK(Beijing) 2020-0004) and fed adaptively for 3 days. A MI model was induced through proximal left anterior descending coronary artery (LAD) ligation as has been described (30, 31), which remains the most acceptable method in rodents to explore the pathophysiology of acute myocardial infarction due to its similarity to humans (32). Simply put, following an intraperitoneal injection of sodium pentobarbital (40 mg/kg) for anesthesia and then intubation, the heart was exposed via a lateral thoracotomy, and finally, the LAD was ligated between the pulmonary cone and left atrial appendage with a 5–0 nylon suture (Shanghai Medical Suture Needle Factory Co., Ltd). Rats in the sham-operated group underwent the same procedure but without ligation. After the surgery, in addition to the sham-operated group (Sham, *n* = 10), the surviving and successfully modeled rats were randomly divided into the model group (MI, *n* = 9) and the Modified Linggui Zhugan Decoction group (MLZD, *n* = 9). All of the animals were housed under the same conditions in a temperature-controlled room (24 ± 1°C) with a natural day/night cycle light and were given ad libitum access to standard chow and water for 4 weeks. All the experimental procedures were approved by the Institutional Animal Care and Use Committee of Guang’anmen Hospital, China Academy of Chinese Medical Sciences, in accordance with the regulations on the management and use of experimental animals.

### 2.3. Preparation of MLZD and interventions

The 10 drugs of the MLZD formula, including Radix astragali (21081961), Panax ginseng (21040161), Ramulus cinnamomic (21092511), Poria (21102131), Rhizoma atractylodis macrocephalae (21100541), Rhizoma alismatis (21090051), Radix salviae miltiorrhizae (21080651), Pericarpium areca (21081271), Semen lepidii (21071351) and Radix angelicae sinensis (21082271), were purchased from Jiangyin Tianjiang Pharmaceutical Co. Ltd. (Jiangsu, China) or Sichuan New Green Pharmaceutical Science and Technology Development Co. Ltd. (Sichuan, China), which met the grade standards of the Chinese Pharmacopoeia. These drugs were provided by the Chinese Pharmacy of Guang’anmen Hospital, China Academy of Chinese Medical Sciences (Beijing, China), and the voucher specimens of all drugs were deposited at the Cardiovascular Laboratory, Guang’anmen Hospital, China Academy of Chinese Medical Sciences (Beijing, China). The clinical dose of MLZD was 190 g, with 30, 10, 10, 30, 15, 30, 20, 15, 15, and 15 g for Radix astragali, Panax ginseng, Ramulus cinnamomi, Poria, Rhizoma atractylodis macrocephalae, Rhizoma alismatis, Radix salviae miltiorrhizae, Pericarpium areca, Semen lepidii, and Radix angelicae sinensis, respectively. In reference to previous studies (33), the MLZD dose was calculated by the equation: Dm = Dh/W × F. Where Dm was the administrated dose of MLZD for rats, Dh was the clinical dose of MLZD, W represented the weight of the human body that was set as 60 kg, and, F was the dose conversion factor that was 6.3 between rats and humans. The drug was dissolved in distilled water and administered at a dose of 19.95 g/(kg day) in this study. The rats were therefore treated with MLZD via gavage once daily starting on the first postoperative day for 4 weeks. Rats in the sham and MI groups were fed equal volumes of physiological saline solution.

### 2.4. Echocardiography and specimen collection

After 28 days of continuous intragastric administration, the cardiac structure, and function of rats were determined by non-invasive transthoracic echocardiography in M-mode, implementing a Vevo-2100 high-resolution echocardiography system (Visual Sonics Inc., Canada). Following being anesthetized with sodium pentobarbital (40 mg/kg), two-dimensional echocardiograms of the left ventricular (LV) long-axis were recorded at the level of the papillary muscle tips for detecting LV ejection fraction (LVEF), LV fractional shortening (LVFS), LV end-diastolic anterior wall thicknesses (LVAW; d), LV end-systolic anterior wall thicknesses (LVAW; s), LV end-diastolic internal diameters (LVID; d), LV end-systolic internal diameters (LVID; s), LV end-diastolic volume (LV Vol; d) and LV end-systolic volume (LV Vol; s). These echocardiographic parameters were obtained by averaging the corresponding parameters of three cardiac cycles.

After echocardiography, the rats were immediately euthanized and their hearts were excised, cut off attachments, irrigated clean with cold saline buffer, measured for weight and size, and transected into two parts at the maximum transverse diameter. The upper part of cardiac tissue was fixed with 4% paraformaldehyde (P1110, Solarbio, CHN) at 4°C for examinations like histopathology and immunohistochemistry, and the apex part was stored at −80°C or fixed with 2.5% glutaraldehyde (P1126, Solarbio, CHN) for western blot analyses, transmission electron microscopy, and the like. The tetramethylrhodamine methyl ester (TMRM) staining should be performed immediately with fresh heart tissue.

### 2.5. Histopathology, immunohistochemistry, and TUNEL

After being fixed in 4% paraformaldehyde overnight, myocardium, liver, and kidney specimens were dehydrated, rendered transparent, embedded with paraffin, and cut into 4-μm-thick transverse sections for hematoxylin and eosin (HE) and Masson staining. The specimens were eventually observed under an optical microscope to assess histopathological changes, and Image J software (National Institutes of Health, USA) was used to quantify the ratio of the blue-positive stained region to the entire surface, which reflected the severity of cardiac fibrosis.

For detecting several crucial proteins through immunohistochemistry, the paraffin slices were boiled in an autoclave for 3 min to repair the antigen. The slices were afterward placed at 60°C for 2 h, deparaffinized, and hydrated with xylene and ethanol, followed by phosphate-buffered saline (PBS) and double-distilled water to wash the retrieved nuclear antigen. Following that, the samples were incubated with primary antibodies overnight at 4°C: SIRT3 antibody (1:200, 2627S, Cell Signaling, USA), anti-mitofusin 2 antibody (10 μg/ml, ab101055, Abcam, UK), phospho-Drp1 (p-Drp1) antibody (1:100, 4867S, Cell Signaling, USA), anti-Bax antibody (1:60, BM3964, BOSTER, CHN), and anti-Bcl-2 antibody (1:500, 26593-1-AP, Proteintech, CHN). The samples were then interacted with HRP-conjugated goat anti-rabbit immunoglobulin G (IgG) at 37°C for 30 min, followed by staining with diaminobenzidine (DAB) detection kit (ZLI-9017, ZSGB-BIO, CHN).

Apoptosis in cardiac tissue was eventually analyzed via terminal deoxynucleotidyl transferase-mediated dUTP nick end labeling (TUNEL) staining using a TUNEL kit (Roche, Switzerland) according to kit protocols. Images were observed under a microscope (Olympus, Japan), and the ratio of apoptosis in randomly selected visual fields was quantified using Image J software (National Institutes of Health, USA).

### 2.6. Transmission electron microscopy

The ultrastructure of cardiomyocytes was observed utilizing a transmission electron microscope (TEM) as has been described (34–36). Briefly, the heart tissues were sectioned into small granules less than 1 mm^3^, fixed in 2.5% glutaraldehyde (P1126, Solarbio, CHN) for 24 h, and then washed in PBS. After that, the tissues were secondary fixed with 1% osmium tetroxide, dehydrated with graded ethanol, embedded in the ultra-thin epoxy part, sliced, uranyl acetate stained, and eventually photographed using a transmission electron microscope (Hitachi, Japan). The mitochondrial numbers and average mitochondrial areas were analyzed and quantified with Image J software.

### 2.7. Cell culture and treatments

H9c2 cells (CL-0089, Procell, CHN) were cultured in Dulbecco’s modified Eagle’s medium (DMEM, 11995, Solarbio, CHN) containing 10% fetal bovine serum (FBS,16000044, Gibco, USA) and 1% streptomycin-penicillin (P1400, Solarbio, CHN) at 37°C in a 5% CO_2_ atmosphere. Oxygen-glucose deprivation (OGD) was performed to emulate the MI model *in vitro* (37, 38). Briefly, cells were incubated with glucose-free and FBS-free DMEM (11966025, Gibco, USA) and low-oxygen incubator (5% CO_2_, 1% O_2_, and 94% N_2_), at 37°C for 4 h. After that, the culture solution was replaced with standard culture media or treated with MLZD, in normoxic conditions for another 24 h. The cells were divided into seven groups: CON, OGD, 3-TYP (1 μM, IT1960, Solarbio, CHN), OGD+3-TYP, MLZD, OGD+MLZD (0.5 mg/ml), and OGD+MLZD+3-TYP. 3-(1H-1,2,3-triazol-4-yl) pyridine (3-TYP) is a selective inhibitor of SIRT3 (39, 40), and its dosage was based on previous studies (40).

### 2.8. Cell viability assay

Cell viability was evaluated with the Cell Counting Kit-8 (CCK-8) (CK04, Dojindo, Japan) according to the manufacturer’s instructions. Briefly, H9c2 were inoculated in 96-well plates at a density of 3,000 cells/well and cultured for 48 h. Then, the cardiomyocytes were treated as described above. Hundred microliter mixture (90 μl DMEM+10 μl CCK8 solution) was then added to each well and incubated at 37°C for 1–4 h. The absorbance was eventually measured with a microplate reader (Rayto, CHN) at 450 nm. Cell viability was expressed as the percentage of OD_450_ values in the control group, which was set at 100%.

### 2.9. Measurement of mitochondrial electron transport chain complex I and IV, and ATP content

Concentrations of cytochrome c oxidase (Complex IV) in cardiomyocytes were determined using a Rat Cytochrome C Oxidase ELISA kit (RJ16349, RENJIEBIO, CHN). The standard substance at known concentrations, cell supernatant and enzyme-conjugate reagents were pipetted into the wells of microplate strips, and incubated for 60 min at 37°C. Then bound enzyme and chromogenic substrate was added successively. Finally, the OD_450_ values were detected that correspond to the complex IV concentration.

According to the manufacturer’s instructions of Mitochondrial Complex I Activity Detection Kit (BC0515, Solarbio, CHN), cells were collected to the extracting solution for homogenization, centrifugation, and ultrasonic crushing. After adding samples and detection reagents into the 96-well plate, the OD_340_ values in 10 s and 2 min were recorded, respectively, as A_1_ and A_2_. Complex I activity (U/mg prot) = 2680 × (A_1_ − A_2_)/Cpr. Where Cpr was the protein concentration of samples.

Based on the instructions of Adenosine Triphosphate (ATP) Chemiluminescence Assay Kit (E-BC-F002, Elabscience, CHN), the cells were collected and mixed in the extracting solution, then bathed in boiling water for 10 minutes, centrifuged, and diluted of the supernatant. After adding enzyme reagents, standard solutions, and samples into the 96-well enzyme label plate, the fluorescence values of each well were measured on the chemiluminescence detector.

### 2.10. Measurement of mitochondrial membrane potential

Mitochondrial membrane potential was detected by tetramethylrhodamine methyl ester(TMRM) staining as described in a previous study (41). Fresh myocardium specimens were cut into 4-μm-thick frozen sections and fixed with 80% ethanol. As for H9c2, they were seeded onto 24-well plates until 80% confluence. After that, the heart tissue or cardiomyocytes were washed with tap water and distilled water successively and then incubated with TMRM at 37°C for 1 h. Fluorescence was subsequently monitored and quantified using laser confocal microscopy and Image J software, respectively. The decreased fluorescence intensity implied mitochondrial membrane depolarization because in normal cells TMRM accumulates in mitochondria and emits bright orange-red fluorescence but the fluorescence weakened significantly when mitochondrial membrane potential decreased.

### 2.11. Western blot analyses

A total of 20 μg of protein extract from myocardial tissues and cardiomyocytes was separated using a 10% Omni-easy™ one-step PAGE gel rapid preparation kit (PG212, Epizyme, CHN) and then transferred to the 0.22 or 0.45 μm PVDF membranes (YA1700/YA1701, Solarbio, CHN). The PVDF membranes were subsequently blocked with 5% nonfat dry milk (9999S, Cell Signaling, USA) at room temperature for 2 h and incubated with primary antibodies at 4°C overnight: anti-SIRT3 antibody (1:1,000, 2627S, Cell Signaling, USA), anti-SOD2/MnSOD antibody (1:2,000, ab16956, Abcam, UK),anti-mitofusin 2 antibody (1 μg/ml, ab101055, Abcam, UK) or anti-mitofusin 2 antibody (1:1,000, bs-2988R, Bioss, CHN), phospho-Drp1 (p-Drp1) antibody (1:1,000, 4867S, Cell Signaling, USA), Drp1 antibody (1:5,000, 12957-1-AP, proteintech, CHN), COX IV antibody (1:1,000, 4850S, Cell Signaling, USA), PGC-1α antibody (1:1,000, ab54481, Abcam, UK), PPAR-γ (1:500, WL01800, Wanleibio, CHN), cytochrome c antibody(1:1,000, 11940S, Cell Signaling, USA), cleaved caspase-3 antibody (1:1,000, 9664S, Cell Signaling, USA), anti-Bax antibody(1:700, WL01637, Wanleibio, CHN), anti-Bcl-2-antibody (1:500, WL01556, Wanleibio, CHN), NLRP3 (1:500, R30750, NSJBIO, USA), ASC (1:1,000, DF6304, Affinity Biosciences, CHN), caspase-1 (1:500, sc-392736, santa cruz, USA), NFκB p50 (1:5,000, ab32360, Abcam, UK), NFκB p65 (1:1,000, ab194726, Abcam, UK) and IL-1β (1:1,000, AF5103, Affinity Biosciences, CHN). We next incubated the PVDF membranes with the secondary antibody HRP-conjugated goat anti-rabbit IgG (H&L) (1:10,000, bs-40295G-HRP, Bioss, CHN) or goat anti-mouse IgG (H&L) (1:1,000, A0216, Beyotime, CHN) at room temperature for 1 h. The gels were finally visualized utilizing Gel Doc™ XR+ System (Bio-Rad, USA) and Image Lab Software (Bio-Rad, USA) with clarity max western ECL substrate (1705062, Bio-Rad, USA), followed by quantitative analysis with Image J software, and the results expressed as density values were normalized to GAPDH or tubulin.

### 2.12. Statistical analyses

Graphpad Prism software (version 8.0) was used for statistical analysis, and the detection was repeated at least three times independently. The data were presented as the mean ± standard deviation($\bar{x}$ s). Comparisons among multiple groups were performed through repeated one-way analysis of variance (ANOVA), with *P* values <0.05 considered to be statistically significant.

## 3. Results

### 3.1. Chemical composition of MLZD

**FIGURE 1:**
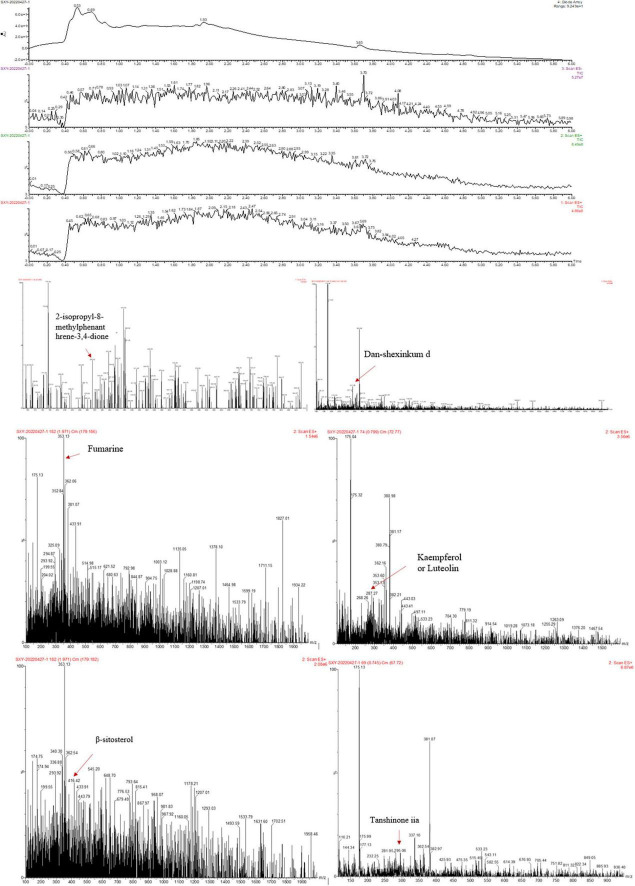
The ingredients of Modified Linggui Zhugan Decoction (MLZD) were identified, and 7 active components were finally obtained.

To preliminarily identify MLZD ingredients and explore their roles in protecting against ventricular remodeling, network pharmacology and LC-MS analysis were implemented to detect the bioactive components of MLZD in this study. Through the TCMSP database, 112 active ingredients were obtained, which are shown in Supplementary Table 1. Based on these components, LC-MS analysis was conducted, and a total of 7 components were finally identified, including 2-isopropyl-8-methylphenanthrene-3,4-dione, dan-shexinkum d, fumarine, kaempferol, luteolin, β-sitosterol, and tanshinone iia. The LC–Mass spectrogram of MLZD after extraction with ultrapure water is shown in Figure 1.

### 3.2. MLZD improved cardiac structure and functions after MI

**FIGURE 2:**
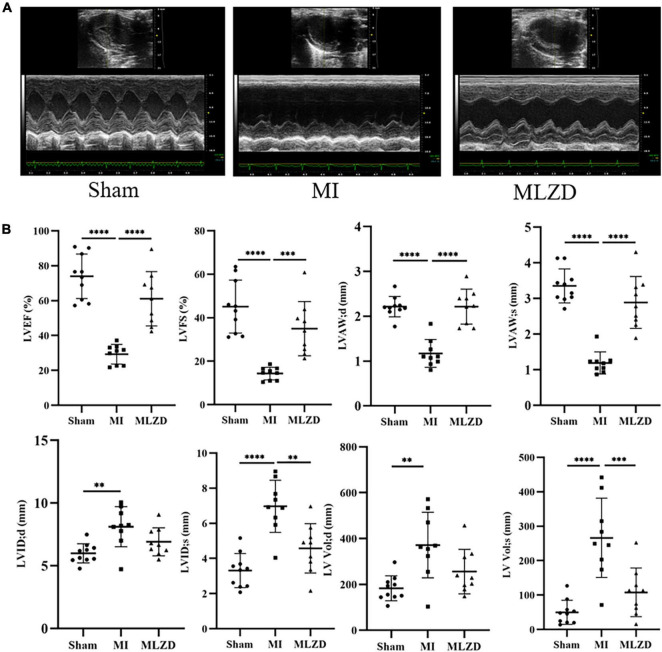
Modified Linggui Zhugan Decoction (MLZD) exerted protective effects on cardiac morphological abnormalities and dysfunctions. **(A)** Representative images of echocardiographic measurements in three groups. **(B)** Quantitative analysis of cardiac structure and function via echocardiography parameters, *n* = 9 or 10 per group. Data are mean ± standard deviation ($\bar{x}$ ± s); ^**^P < 0.01, ^***^P < 0.001, ^*⁣***^P < 0.0001.

We first implemented echocardiography and hemodynamic analyses to monitor the establishment of MI models induced by LAD ligation and the influences exerted by MLZD on cardiac structure and function. Representative two-dimension echocardiograms are shown in Figure 2A and comparisons of corresponding parameters among three groups are illustrated in Figure 2B. Four-week disposal-free feeding following LAD ligation produced significant remodeling manifestations, evidenced by augmentation in LVID;d, LVID;s, LV Vol;d, and LV Vol;s. Other structural changes are represented by decreased LVAW;d and LVAW;s. Furthermore, worse cardiac function was found in rats of the MI group than that of the sham group, according to deductions in LVEF and LVFS. However, all these parameters were improved to some extent after MLZD treatment versus the MI group, and no significant difference was exhibited between the sham and MLZD groups.

### 3.3. MLZD ameliorated cardiac fibrosis and remodeling

**FIGURE 3:**
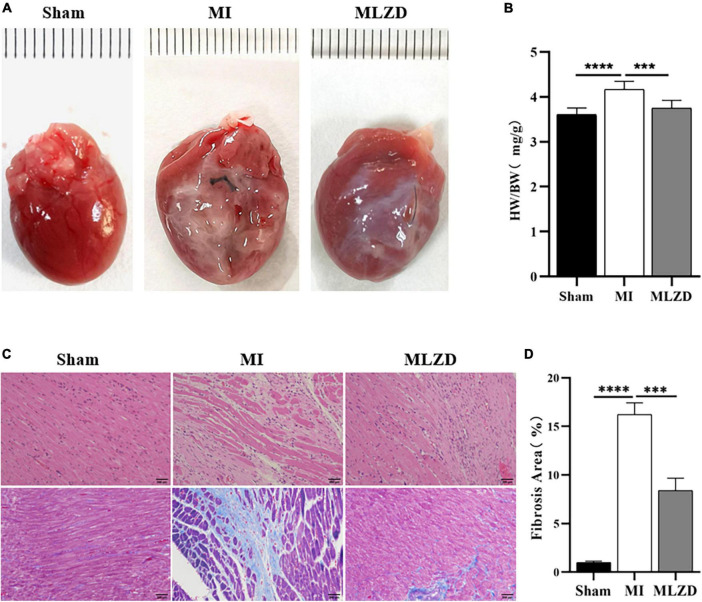
Modified Linggui Zhugan Decoction (MLZD) ameliorated cardiac fibrosis and reversed ventricular remodeling. **(A)** The appearance of hearts in three groups. **(B)** Ratios of heart weight to total body weight. **(C)** Representative images of HE and Masson trichrome staining, scale bar = 200 μm. **(D)** Quantification of myocardial fibrosis by fibrosis area, *n* = 3 per group. Data are presented as mean ± standard deviation ($\bar{x}$ ± s); ^***^P < 0.001, ^****^P < 0.0001.

The heart appearance and results of heart weight to total body weight ratios (HW/BW) revealed slightly enlarged hearts in MI rats and improved conditions after MLZD intervention (Figures 3A, B). Through HE staining of heart sections from rats in each group, destroyed cardiomyocyte arrangement and inflammatory infiltration were observed in the infarcted hearts. Furthermore, the quantification of heart fibrosis exhibited more collagen deposition and enlarged fibrosis areas in model rats, which were evidenced by increased blue region. These histopathological injuries were alleviated following MLZD treatment to some extent, maintaining cardiomyocyte integrity and relieving myocardial fibrosis (Figures 3C, D). These data suggest that MLZD reversed cardiac fibrosis and remodeling exacerbated following MI.

### 3.4. MLZD inhibits mitochondrial-associated apoptosis

**FIGURE 4:**
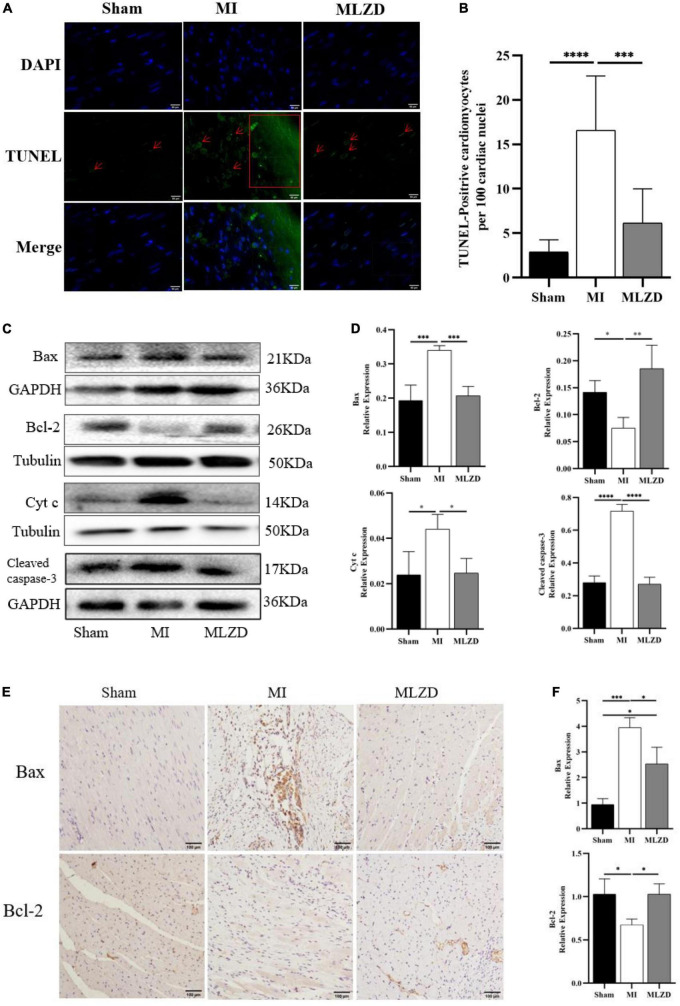
Modified Linggui Zhugan Decoction (MLZD) ameliorated apoptosis. **(A)** TUNEL staining for heart tissue, scale bar = 50 μm. **(B)** Quantitative analysis for apoptosis, *n* = 6 or 8 per group. **(C,D)** Immunoblot analyses of Bax, Bcl-2, Cyt c, and cleaved-caspase-3 in the hearts of different groups. The widths of the images have been compressed at a ratio of 1:0.6. *n* = 4 or 6 per group. **(E,F)** Immunohistochemical analyses for detecting Bax and Bcl-2 expression, *n* = 3 per group, scale bar = 100 μm. Data are represented as mean ± standard deviation ($\bar{x}$ ± s); *P < 0.05, ^**^P < 0.01, ^***^P < 0.001, ^****^P < 0.0001.

To further verify MI-induced myocardial damage and the role of MLZD in this process, we examined whether apoptosis occurred and its related pathways. As shown by results for TUNEL staining in the heart tissue (Figures 4A, B), the apoptosis rate in infarcted myocardium increased, while it was ameliorated following MLZD intervention. Consistently, the expression of the mitochondria-associated apoptotic proteins was altered in the hearts of rats (Figures 4C–F). The results were shown with increased levels of Bax, Cyt c, and cleaved caspase-3 but decreased Bcl-2 in the MI group, while the expression levels were reversed in the drug intervention group.

### 3.5. MLZD ameliorates myocardial inflammation

**FIGURE 5:**
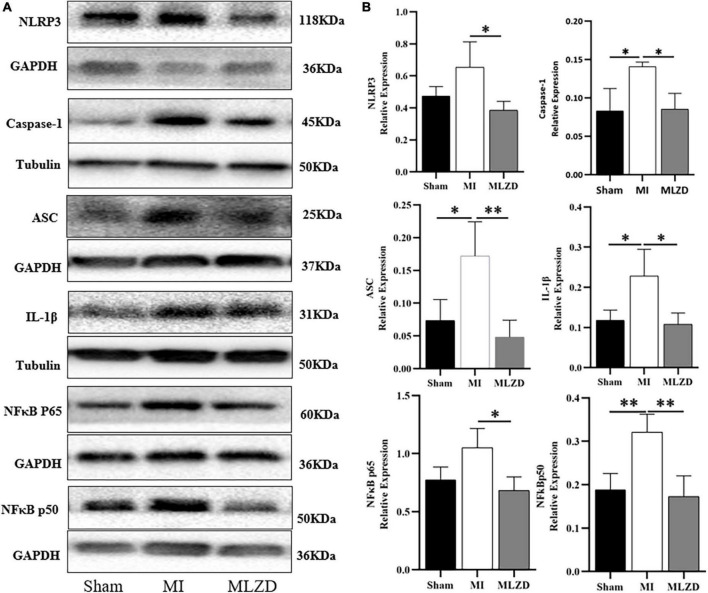
Modified Linggui Zhugan Decoction (MLZD) inhibits the expression of inflammatory proteins. **(A,B)** Western blot analyses of the expression of NLRP3, caspase-1, ASC, IL-1β, NFκB p65, and NFκB p50 in cardiac tissue. The widths of the images have been compressed at a ratio of 1:0.6. *n* = 3 or 4 per group. Data are represented as mean ± standard deviation ($\bar{x}$ ± s); *P < 0.05, ^**^P < 0.01.

To verify the regulating effect of MLZD on myocardial inflammation, inflammation-related proteins including NLRP3, caspase-1, ASC, IL-1β, NFκB p65, and NFκB p50 in the myocardial tissue of rats in three groups were detected. As shown in Figure 5, the expression level of the above inflammatory proteins was increased in the MI group, but improved after drug administration.

### 3.6. MLZD repairs mitochondrial damage

**FIGURE 6:**
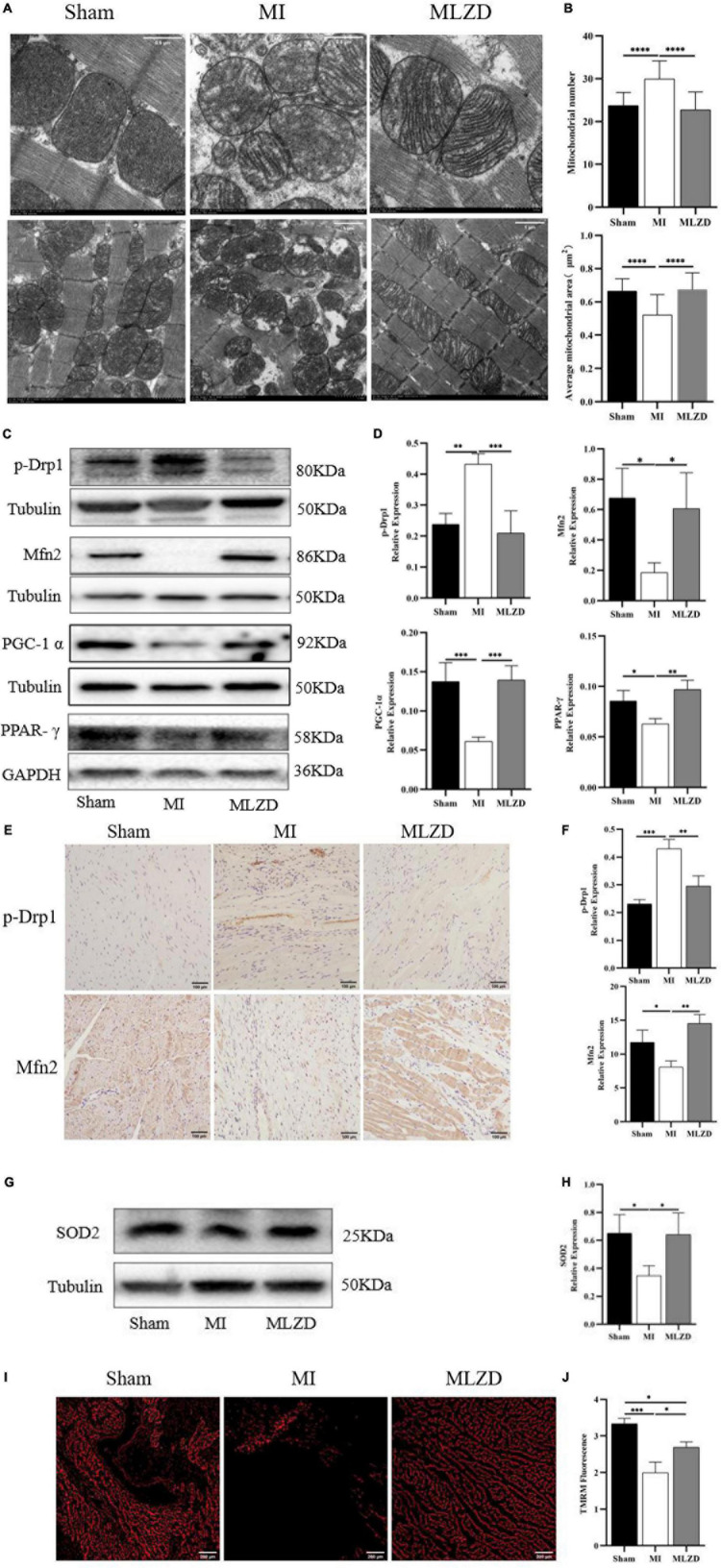
Modified Linggui Zhugan Decoction (MLZD) repairs mitochondrial damage. **(A)** The representative transmission electron microscope (TEM) images of mitochondria in the hearts at a magnification of 8.0k and 3.0k, respectively (Scale bar = 0.5 or 1 μm). **(B)** The mitochondrial number changes and the average mitochondrial area (μm^2^) under TEM in heart tissue, *n* = 20 visual fields (5 visual fields per rat, 4 rats per group). **(C,D)** Western blot analyses of the expression of p-Drp1, Mfn2, PGC-1α, and PPAR-γ in cardiac tissue. The widths of the images have been compressed at a ratio of 1:0.6. *n* = 4 per group. **(E,F)** Relative expression of p-Drp1 and Mfn2 in heart detected through immunohistochemical analyses, *n* = 3 per group, scale bar = 100 μm. **(G,H)** Western blot analyses of SOD2 expression in the heart. The widths of the images have been compressed at a ratio of 1:0.6. *n* = 4 per group. **(I)** Representative images of TMRM staining captured via fluorescent microscopy, scale bar = 200 μm. **(J)** Quantitation of fluorescence intensity in different groups, *n* = 3 per group. Data are mean ± standard deviation ($\bar{x}$ ± s); *P < 0.05, ^**^P < 0.01, ^***^P < 0.001, ^****^P < 0.0001.

To examine the effects of MLZD on mitochondrial morphology and dynamics changes, mitochondrial ultrastructure was observed through an electron microscope, and Mfn2, p-Drp1, PGC-1α, and PPAR-γ expression were detected by western blot or immunohistochemistry. Intact outer membrane and dense cristae of mitochondria were mainly shown in the normal group, while more abnormal mitochondrial morphologies were provoked in the MI rats along with decreased mitochondria volume, disarrayed cristae, and swollen matrix. The MLZD treatment gradually recovered the mitochondrial structure (Figure 6A). Further quantitative analyses showed that MLZD significantly decreased the average numbers of mitochondria but significantly increased their average sizes (Figure 6B). Moreover, disturbed expression of Mfn2 and p-Drp1, and decreased levels of PGC-1α and PPAR-γ were induced by MI but substantially reversed via MLZD administration (Figures 6C–F). Superoxide dismutase 2 (SOD2) detection and TMRM staining were further performed to assess mitochondrial antioxidant capacity and MMP. The level of SOD2, along with MMP, decreased in the MI group compared with the sham group but improved following MLZD treatment (Figures 6G–J).

### 3.7. MLZD might achieve mitochondrial protection via SIRT3

**FIGURE 7:**
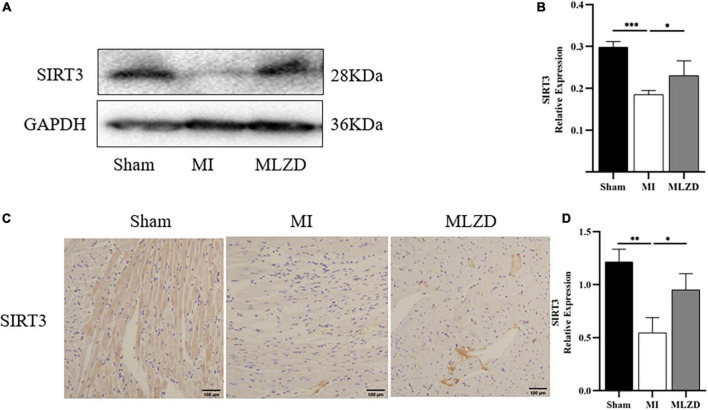
Modified Linggui Zhugan Decoction (MLZD) promoted SIRT3 expression in cardiac tissue. **(A,B)** Relative expression of SIRT3 detected via immunoblot analyses. The widths of the images have been compressed at a ratio of 1:0.6. *n* = 4 per group. **(C,D)** Immunohistochemical analysis for detecting SIRT3, *n* = 3 per group, scale bar = 100 μm. Data are represented as mean ± standard deviation ($\bar{x}$ ± s); *P < 0.05, ^**^P < 0.01, ^***^P < 0.001.

To clarify the possible pathways by which MLZD ameliorates mitochondrial abnormalities, the relative protein expression of SIRT3 in the cardiac tissue was detected considering its regulation of mitochondria. The results revealed the downregulated generation of SIRT3 in the MI rats compared with the healthy ones, while treatment of MLZD blocked the effect (Figure 7). These results suggested that SIRT3 could be one of the targets of MLZD in mitochondrial oxidative stress, mitochondrial dynamics disorder, MMP decrease, mitochondria-induced apoptosis, and even the pathological process of ventricular remodeling.

### 3.8. 3-TYP blocked the effects of MLZD on promoting SIRT3 expression and protecting cardiomyocytes

**FIGURE 8:**
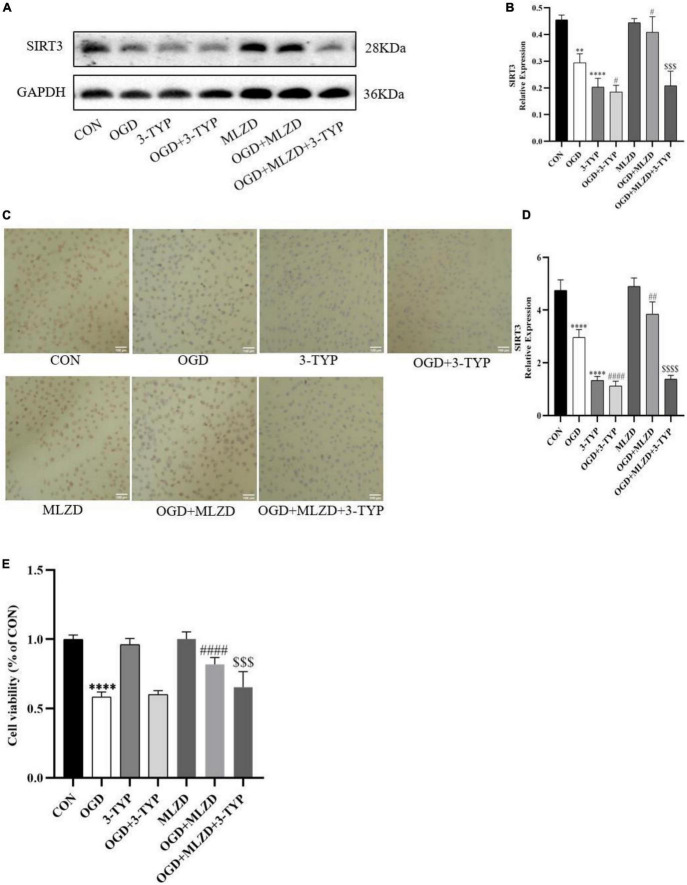
3-TYP inhibited SIRT3 expression and prevented the cardiomyocyte protective effect of MLZD. **(A,B)** Relative expression of SIRT3 in cardiomyocytes detected via western blot. The widths of the images have been compressed at a ratio of 1:0.8. **(C,D)** Immunohistochemical analysis for detecting SIRT3, scale bar = 100 μm. **(E)** The cell viability in seven groups evaluated through the CCK-8 test kit. Data are represented as mean ± standard deviation ($\bar{x}$ ± s); ^**^P < 0.01, ^****^P < 0.0001 vs. CON; ^#^*P* < 0.05, ^##^*P* < 0.01, ^####^*P* < 0.0001 vs. OGD; ^$$$^*P* < 0.001, ^$$$$^*P* < 0.0001, vs. OGD+MLZD.

To determine the role of SIRT3 in MLZD-mediated cardioprotection, H9c2 was treated with 3-TYP, a selective inhibitor of SIRT3. The expression level of SIRT3 and cell viability of treated cardiomyocytes were detected. As shown in Figures 8A–D, MLZG stimulated SIRT3 expression in OGD cells, but its effect was blocked by 3-TYP. In addition, 3-TYP attenuated MLZD-mediated cardiomyocyte protection (Figure 8E). To exclude the specific contribution of the single elements, the effects of 3-TYP on normal or OGD cardiomyocytes were evaluated, and the results showed that 3-TYP had no additional effects except inhibiting SIRT3. According to MLZD vs. CON, in addition, MLZD showed no significant adverse effects.

### 3.9. 3-TYP inhibited the protective effects of MLZD on mitochondria

**FIGURE 9:**
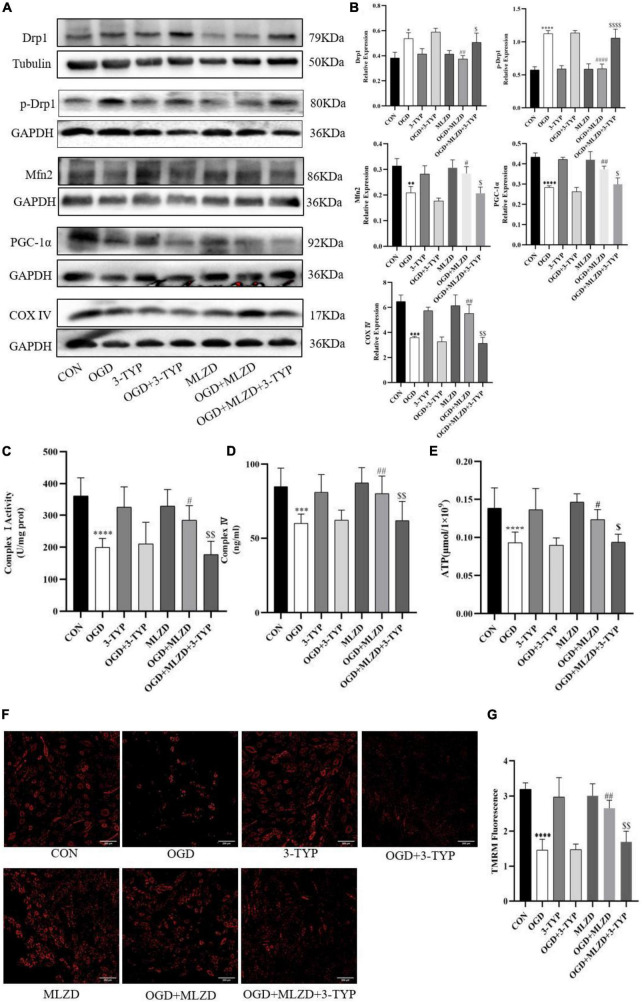
3-TYP reversed the regulation of mitochondria by Modified Linggui Zhugan Decoction (MLZD). **(A,B)** Detection of Drp1, p-Drp1, Mfn2, PGC-1α, and COX IV in cardiomyocytes by western blotting. The widths of the images have been compressed at a ratio of 1:0.8. **(C,D)** Detection of mitochondrial electron transport chain complex I and IV in cardiomyocytes. *n* = 8 per group. **(E)** Determination of ATP content in cardiomyocytes. *n* = 8 per group. **(F,G)** TMRM staining for assessing mitochondrial membrane potential, *n* = 3 per group, scale bar = 200 μm. Data are represented as mean ± standard deviation ($\bar{x}$ ± s); *P < 0.05, ^**^P < 0.01, ^***^P < 0.001, ^****^P < 0.0001 vs. CON; ^#^*P* < 0.5, ^##^*P* < 0.01, ^####^*P* < 0.0001 vs. OGD; ^$^*P* < 0.05, ^$$^P < 0.01, ^$$$$^*P* < 0.0001 vs. OGD + MLZD.

The effects of MLZD on mitochondria in OGD cells and the role of 3-TYP in this process were evaluated by detecting Drp1, p-Drp1, Mfn2, PGC-1α, cytochrome c oxidase subunit 4 (COX IV), mitochondrial electron transport chain complex I and IV, ATP content, and MMP. The results showed that in OGD model cells MLZD alleviated increased Drp1 and p-Drp1 expression, while it promoted Mfn2, PGC-1α, and COX IV expression. However, this effect was reversed by the SIRT3 inhibitor 3-TYP (Figures 9A,B). And 3-TYP additionally blocked the protective effects of MLZD on complex I and IV, ATP, and MMP (Figures 9C–G). Furthermore, in the present study 3-TYP and MLZD showed no obvious adverse effects on normal or OGD cardiomyocytes.

## 4. Discussion

Due to a lack of effective interventions, MI and MI-caused ventricular remodeling have become serious health threats worldwide. As a complementary and alternative medicine, TCM has great potential to alleviate this health burden. In this pharmacological study through in vivo experiments in rats, we found that MLZD protected against post-MI ventricular remodeling, in part by alleviating mitochondrial injury and mitochondrial-associated apoptosis. Further experimental verification revealed that the cardioprotective mechanism of MLZD may be exerted partly by promoting SIRT3 expression.

The active components of 10 TCM in MLZD were analyzed by the TCMSP database and LC-MS analysis. A total of 7 compounds were identified, including 2-isopropyl-8-methylphenanthrene-3,4-dione, dan-shexinkum d, fumarine, kaempferol, luteolin, β-sitosterol, and tanshinone IIa. Studies have shown that kaempferol exerts protective effects on cardiac/cardiomyocyte injury by inhibiting inflammation mediated by STING/NF-κB (42), regulating miR-15b/Bcl-2/TLR4 (43), and through some other mechanisms. Luteolin, a falconoid compound, can exert myocardial ischemia protection by reducing MI area, apoptosis, and inflammation (44). Luteolin may additionally alleviate doxorubicin-induced cardiotoxicity, including apoptosis, ROS accumulation, and mitochondrial membrane potential collapse (45). β-Sitosterol may alleviate cardiac necrosis and apoptosis by inhibiting inflammatory responses and oxidative stress (46). In the animal experiments to test the safety of the drug, there was no significant change in weight and mortality of rats treated with MLZD, and no obvious pathological changes were found by HE staining of liver and kidney tissues (Supplementary Figure 1). *In vitro* experiments, additionally, we first examined the viability of H9c2 cardiomyocytes treated with MLZD alone without OGD, and subsequently tested the effects of MLZD on OGD, finding that MLZD had a protective effect on cell viability (Supplementary Figure 2). Therefore, in the current study, by referring to similar grouping schemes (17), the rats were divided into sham, MI, and MLZD groups, not setting the group receiving MLZD without MI.

Cardiac dysfunction involving MI triggers maladaptive myocardial responses—including fibrosis, wall thinning, and ventricular dilation—which contribute to post-infarction myocardial remodeling, thereafter leading to impaired contractile function, and eventually heart failure (5, 47). Ventricular remodeling mainly results from the reduction of cardiomyocytes and the undesirable development of surviving cardiac cells and extracellular matrix (5). Ventricular myocyte fibrosis is deemed as the characteristic appearance of cardiac hypertrophic remodeling and is closely related to heart failure (17). After 4 weeks of intervention, the results of echocardiography, histological analysis, and quantitative fibrosis analysis indicated that the deterioration of cardiac structure and function, histopathological changes, and fibrosis caused by MI were partially reversed by MLZD.

Apoptosis may cause infarction extension (48), cardiac remodeling (49), cardiac dysfunction, and even heart failure (50, 51). The Bcl-2 family plays a crucial part in the promotion or inhibition of the intrinsic apoptotic pathway triggered by mitochondrial dysfunction (52). And the relative expression levels of Bcl-2 and Bax determine cell fate after apoptotic stimulation (53). Beyond that, cytochrome c (Cyt c) and cleaved caspase-3 are also important indicators of apoptosis (54, 55). Specifically, rupture of the outer mitochondrial membrane leads to Cyt c release from the intermembrane space and subsequent inner mitochondrial membrane depolarization (56). At the same time, mitochondrial membrane potential(MMP) decrease, in turn, leads to Cyt c release (57). In the cytoplasm, apoptosomes formed with Cyt c, apoptotic protease-activating factor 1 (Apaf-1), and caspase-9 trigger caspase-3 activation, ultimately leading to apoptosis (10, 58). Bcl-2 blocks cytochrome c and apoptosis-inducing factor release (59). Bax, conversely, increases the permeability of the outer mitochondrial membrane and promotes the release of apoptotic factors (60–62). From the results in Figure 4, MLZD alleviates mitochondrial-associated apoptosis and regulates the expression of apoptosis-related proteins to near-physiological levels. Apoptosis is a process of programmed cell death, representing a critical pathway for eliminating unnecessary and significantly damaged cells, and causes disease when miscontrolled (63, 64). Based on the report that apoptosis is critical to the morphogenesis of the human cardiac conduction system and redintegration of the right ventricle (65) and that, what’s more, myocytes are just a subset of cells in the heart (66), these may explain the high rates of TUNEL positivity and cleaved caspase-3 in normal myocardial tissue of the sham group. A similar pattern has been found in other reports (67, 68).

The NLRP3 inflammasome, a multiprotein binding compound consisting of NLRP3, connector protein ASC and effector protein pro-caspase-1, is activated under stress and plays an important role in cardiac fibrosis (69–71). The inflammasome exerts an inflammatory effect by regulating the release of proinflammatory cytokines including IL-1β and IL-18, contributing to cardiomyocyte apoptosis and dysfunction, and leading to ventricular remodeling and heart failure (69, 72, 73). NFκB is a master regulator of inflammatory gene expression and is activated in a variety of cardiac diseases, including congestive heart failure and cardiac hypertrophy (74). More and more reports have shown that the NFκB pathway plays an important role in the regulation of NLRP3 inflammasome (74–76). In the present study, we demonstrated that MLZD applied to MI rats can inhibit the expression of the above inflammatory proteins.

Mitochondria are the major source of pro-apoptotic factors (77). Mitochondria are most sensitive to ischemia and hypoxia, which generally first damage mitochondrial structure and function (78). Mitochondrial dysfunction is deemed a precursor to cell death (79), and the core reason for heart failure progression (6). Among the many mechanisms that influence mitochondrial stability, mitochondrial dynamics is crucial to cell quality control and function (80, 81) and its disturbance is one cause of apoptosis (82). For instance, mitochondrial fusion promotes structural and functional stability of the inner membrane, thus protecting cells from apoptosis, while fission is related to cell apoptosis (36). Mitochondrial fission alters the outer mitochondrial membrane permeability, resulting in Cyt c release into the cytoplasm, which activates the caspase pathway in a permanent manner and eventually causes apoptosis (83). Besides, mitochondrial damage also activates the NLRP3 inflammasome and causes cell death through ROS overproduction, MMP collapse, and other processes (72, 84, 85). Unbalanced mitochondrial dynamics, inclined to fission and fragmentation, were found in the models of myocardial injury and heart failure (86–88). Among many regulatory proteins, Mfn2 and Drp1 are involved in mitochondrial fusion and mitochondrial fission, respectively (89, 90). Peroxisome proliferator-activated receptor-γ coactivator-1α (PGC-1α), a powerful transcription factor, is the major regulator of mitochondrial biogenesis due to its regulatory effect on processes like energy metabolism and dynamics (91). It functions as a crucial regulator of mitochondrial fusion and fission mainly by influencing Mfn1, Mfn2, and DRP1 and thus maintaining the stabilization of the mitochondrial network (92). PGC-1α is also significant for apoptosis inhibition and, as reported, increased Bcl-2 levels but decreased Bax, cleaved caspase-3, and apoptotic DNA fragmentation were shown in the presence of PGC-1α (93, 94). PGC-1α is a coactivator of peroxisome proliferator-activated receptor (PPAR)-γ (12), and additionally, PPAR deletion significantly decreases PGC-1 expression, thereby leading to mitochondrial structural damage and dysfunction (13). Previous reports suggest that PPAR-γ activation increased MMP and protected cells from apoptosis (95). Mitochondrial electron transport chain complex I and IV, COX IV content, along with ATP, are regarded as markers of mitochondrial function (96, 97), and COX IV is a classic enzyme marker of electron transport chain (97). In the present experiment, abnormal mitochondrial morphologies, increased mitochondrial numbers, decreased mitochondrial areas, disturbed expression of Mfn2, p-Drp1 or Drp1, PGC-1α, PPAR-γ, and COX IV induced by MI or OGD, were ameliorated through MLZD administration. Studies have shown that ROS formation induces the opening of the MPTP and the destruction of MMP, resulting in the subsequent increase of Cyt c, followed by a succession of caspase cascades, and finally apoptosis (98). Additionally, high levels of ROS induce MMP depolarization, triggering mitochondrial fragmentation, and shortening and thus promoting mitochondrial fission (99, 100). SOD2, the key mitochondrial antioxidant enzyme to eliminate free radicals, is located in the mitochondrial matrix following transcription and synthesis and is endowed with the responsibility of converting superoxide to hydrogen peroxide (101, 102). According to the results of Figure 6, the activity of SOD2 was suppressed by ischemia, which was consistent with previous studies (101), but improved to some extent following treatment with MLZD. MLZD could, additionally, inhibit the collapse of MMP in the hearts of rats subjected to MI.

Sirtuin 3 (SIRT3) is a prominent deacetylase mainly found in mitochondria and influences almost all the main aspects of mitochondrial function (103). It is involved in mitochondrial metabolism, redox balance, and mitochondrial dynamics through governing mitochondrial protein acetylation, thereby exerting protective effects against mitochondrial damage (6). It was demonstrated to deacetylate the mitochondrial SOD2, reducing ROS generation and mitochondrial fragmentation (101). Moreover, SIRT3 also regulates the opening of the MPTP through CyPD deacetylation, thereby inhibiting mitochondrial swelling and rupture under stress, maintaining mitochondrial morphology and function, and preventing cardiomyocyte apoptosis and compensatory hypertrophy of residual cardiomyocytes (104). As for MLZD, various components hold great potential for SIRT3 activation. For example, ginsenoside Rg3, a bioactive ingredient of Panax ginseng, ameliorates mitochondrial dysfunction and apoptosis through the SIRT1/PGC-1α/SIRT3 pathway (105); long-term consumption of ginseng extract exerted protective effects on intermediate-aged hearts in rats, which might be mediated partly through the upregulation of SIRT3 (106); Kaempferol could not only increase SIRT3 gene expression but also promoted the expression of deacetylase SIRT3 in the mitochondria (107); Kaempferol could additionally alleviate H9c2 cardiomyocyte ischemia/reperfusion injury through the activation of SIRT3 to inhibit oxidative stress (108); In researches investigating the role of luteolin in cerebral ischemia-reperfusion and ultraviolet radiation B-induced photoaging, luteolin has been reported to exert cellular protection by targeting and promoting SIRT3 (103, 109). In the current study, 3-TYP, the SIRT3 inhibitor 3-TYP blocks the protective effect of MLZD on cardiomyocytes and mitochondria. Therefore, MLZD may protect hearts by targeting SIRT3 to inhibit mitochondrial oxidation, regulate mitochondrial dynamics, and improve mitochondrial structure and function abnormalities. Even though the inhibition of 3-TYP on SIRT3 expression and its adverse effects on cells or animals have been widely reported (39, 110–113), it is noteworthy that a few studies have shown that 3-TYP can only inhibit the activity of SIRT3 but not affect its expression (114, 115). This may be due to the difference in the study subjects, the effects of the combined drugs, the effects of the dose and duration of 3-TYP, or some other factors. The absence of concentration and time gradient, other SIR3 inhibitors and in vivo blocking of SIRT3 is a limitation of our results.

## 5. Conclusion

**FIGURE 10:**
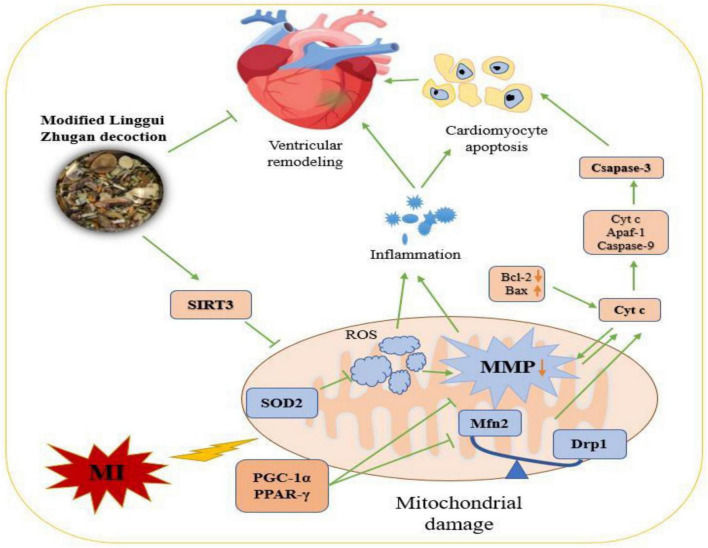
The mechanisms of Modified Linggui Zhugan Decoction (MLZD) in ventricular remodeling of left anterior descending coronary artery ligation-induced myocardial infarction models. Modified Linggui Zhugan Decoction (MLZD) intervention could exert protective effects on mitochondria, including enhancing mitochondrial antioxidant capacity, regulating mitochondrial dynamics disorder, and inhibiting mitochondrial membrane potential collapse, which contributed to preventing mitochondrial-mediated cardiomyocyte apoptosis. The heart-protective effect of MLZD might associate with SIRT3 activation in the cardiac tissue. MI, myocardial infarction; MMP, mitochondrial membrane potential; SIRT3, sirtuin 3; SOD2, superoxide dismutase 2; ROS, reactive oxygen species; Mfn2, mitofusin2; Drp1, dynamin-related protein 1; PGC-1α, peroxisome proliferator-activated receptor-γ coactivator-1α; Cyt c, cytochrome c; PPAR-γ, peroxisome proliferator-activated receptor – γ; Bcl-2, B cell lymphoma-2; Bax, Bcl-2-associated X; Apaf-1, apoptotic peptidase activating factor 1.

Evaluating the collective evidence, it indicated that MLZD treatment could ameliorate post-myocardial infarction ventricular remodeling by inhibiting apoptosis induced by mitochondrial abnormalities. This research described a promising therapy for MLZD, indicating that it prevented apoptosis by protecting mitochondria, including ameliorating mitochondrial structural disruption, protecting against mitochondrial dynamics disorder, restoring impaired mitochondrial function and inhibiting inflammation, which may be exerted by promoting SIRT3 expression (Figure 10). MLZD might therefore represent a new therapeutical possibility for ventricular remodeling and even heart failure following MI, despite the need for further work.

## Data availability statement

The original contributions presented in this study are included in the article/Supplementary material, further inquiries can be directed to the corresponding authors.

## Ethics statement

This animal study was reviewed and approved by Institutional Animal Care and Use Committee of Guang’anmen Hospital, China Academy of Chinese Medical Sciences.

## Author contributions

MX wrote the main text. XZ, YL, YZ, FD, LLv, YW, and ZS retrieved and organized the documents. XC and LLi made great contributions to revisions, polishing this manuscript, and with revising figures. All authors contributed to the article and approved the submitted version.

## Footnotes

1
https://old.tcmsp-e.com/tcmsp.php
