# Supplementary figures and images for: Modified Linggui Zhugan Decoction protects against ventricular remodeling through ameliorating mitochondrial damage in post-myocardial infarction rats
Source: Front Cardiovasc Med. 2023 Jan 10;9:1038523. doi: 10.3389/fcvm.2022.1038523 (PMC9872118; doi:10.3389/fcvm.2022.1038523)

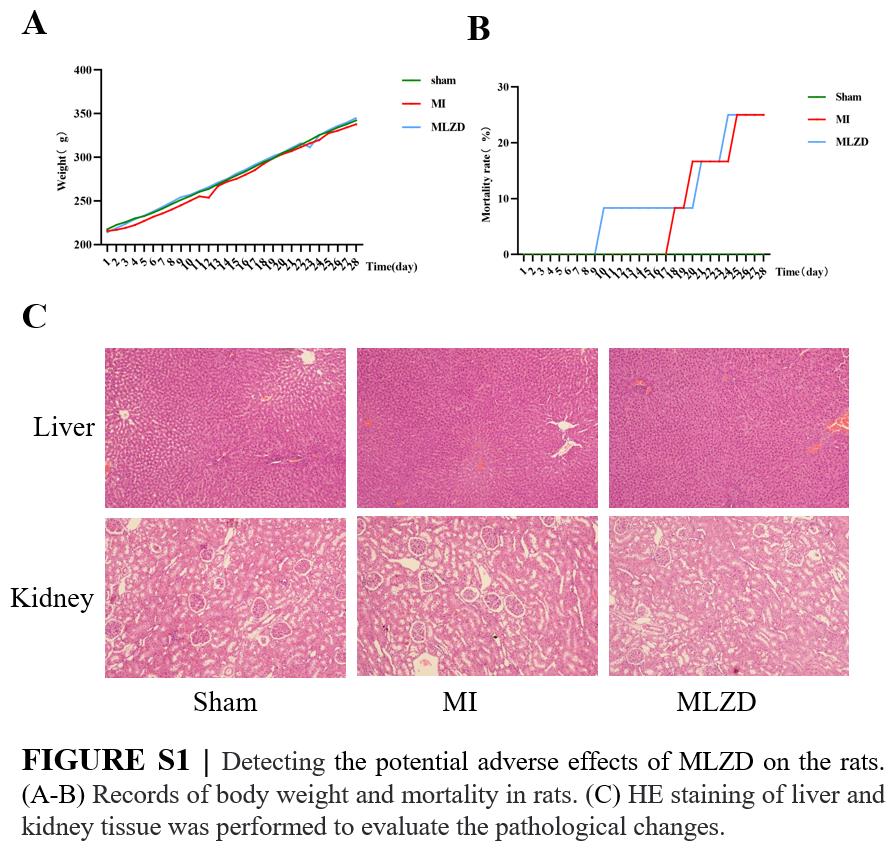

Supplement: Supplementary file 1 [file Image_1.jpeg]

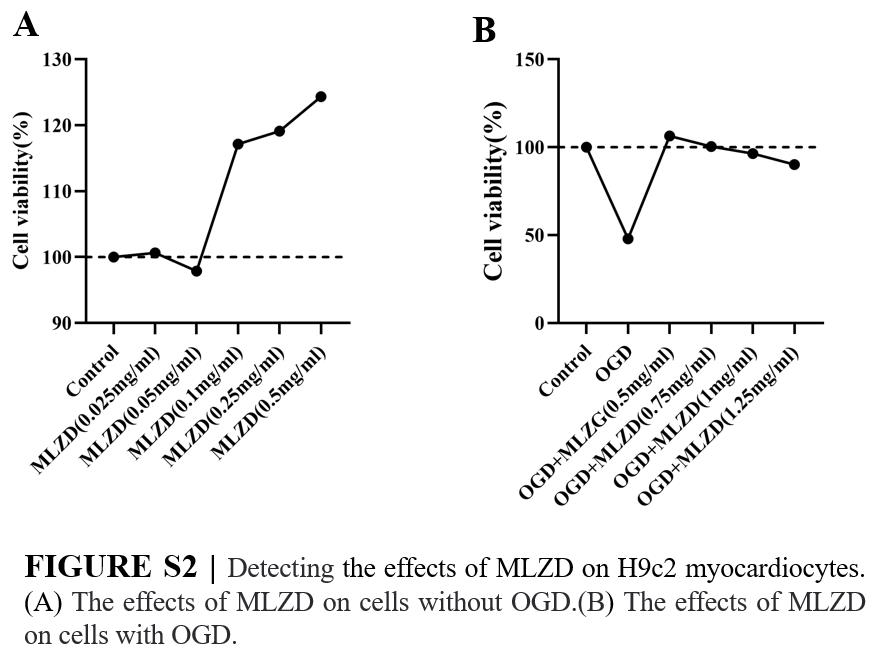

Supplement: Supplementary file 2 [file Image_2.jpeg]
